# Design of a Low-Reflection Flat Lens Antenna Based on Conformal Transformation Optics

**DOI:** 10.3390/mi14030558

**Published:** 2023-02-27

**Authors:** Fateme Nazarzadeh, Abbas Ali Heidari

**Affiliations:** Department of Electrical Engineering, Yazd University, Yazd 8915818411, Iran

**Keywords:** flat lens antenna, transformation optics (TO), conformal transformation optics (CTO), isotropic, graded-index lens

## Abstract

In this paper, a wideband flat lens antenna with low reflection and good performance is presented based on conformal transformation optics (CTO). Physical space optimization is applied to eliminate singular refractive index values. Furthermore, we employ the optical path rescaling method to enhance the sub-unity refractive indices and to reduce reflection. Therefore, an implementable all-dielectric isotropic medium is obtained. The final flat lens profile comprises six layers with a constant permittivity value in each layer. Simulation results of the three-dimensional structure indicate that the designed flat lens operates in a wide frequency bandwidth. The flat lens antenna has an S_11_ value of less than −15 dB in the frequency range of 13 to 30 GHz. The proposed lens was designed and simulated using COMSOL Multiphysics, and radiation performance results were validated using the CST Studio Suite. The simulated radiation pattern shows that the side lobe level is less than −16.5 dB in two simulation software programs, and the half-power beam width varies from 5.6° to 2.7° with increasing frequency. Moreover, the simulated antenna gain is about 28.3–35.5 dBi in the 13–30 GHz frequency range.

## 1. Introduction

Transformation optics (TO) is a theory that can arbitrarily control electromagnetic waves using the invariance form of Maxwell’s equations under a coordinate transformation [1]. This attribute indicates that the form of Maxwell’s equations in different coordinates remains unchanged if the electric permittivity and magnetic permeability tensors are represented as a function of space coordinates. Pendry and Leonhardt proposed the TO concept for the first time in 2006 [2,3]. However, it has since been extensively studied by researchers while designing other devices, such as couplers [4,5,6], flat reflector antennas [7,8,9,10], polarization splitters [11,12,13], concentrators [14,15], and beam bending structures [16,17]. Generally, the materials resulting from transformation optics are complicated and challenging for practical realization. These materials are frequently inhomogeneous, anisotropic, and have unusual permittivity and permeability values, which are often created by resonant metamaterials and limit the operating frequency bandwidth. On the other hand, all-dielectric graded-index materials (GRIN) are beneficial in device realization and can operate over a broad bandwidth [18,19].

The conformal TO (CTO) and quasi-conformal TO (QCTO) approaches are used to overcome the limitation of anisotropy, achieve a GRIN media, and enable the fabrication of devices with isotropic all-dielectric materials. Limiting the coordinate transformation to CTO results in isotropic constitutive parameters and simpler implementation [3,20]. Furthermore, QCTO reduces anisotropy rather than eliminating it [21]. Thus, CTO (QTCO) is employed to address the anisotropic medium problems. However, the inhomogeneous property of the materials is challenging. Dielectric drilling [22], lithography [23], and graded photonic crystals [24] are three methods for solving the limitation of inhomogeneous properties in dielectric graded-index media.

Lens antennas have attracted researchers’ attention because of their wide bandwidth and high gain. Transmitarray antennas have recently been proposed as a replacement for lens antennas, but their main limitation is their narrow frequency bandwidth [25]. The transformation optics theory may be applied to lens design. In recent years, many lenses have been proposed using TO [19,26,27,28,29,30,31,32,33,34,35,36]. The design’s primary mechanism is based on mapping cylindrical phase fronts of the excitation to the phase fronts of a plane wave. Some designs are based on the general form of transformation optics, and the resulting materials are quite complex and challenging to implement [26,27]. QCTO and CTO can be employed to create graded index lenses for a given excitation with an arbitrary phase front [28,29,30,31,32,33,34,35,36]. However, using these approaches in previous research has been associated with shortcomings. The refractive index of the lenses lacks matching to free space at the aperture because mapping a curved phase front line to a straight one results in a non-unity refractive index in the lens’s boundaries. Therefore, designed lenses have a reflection at the boundary [19,26,27,28,29,30,31,32,33,34,35,36]. Furthermore, reflections exist on both sides of the lens if the source is not embedded in the transformed structure [33,36]. In general, reflection from the boundary is present in most designed lenses based on transformation optics. The virtual space is stretched to the physical space form in some parts, so the refractive index goes below unity, and these regions frequently take up much space from the lens. Furthermore, this stretching can occur at the lens’s boundaries and cause reflections. Metamaterials can be employed to realize permittivities less than one. However, the frequency bandwidth is limited due to their resonance structures. Moreover, the unusual refractive index can appear at sharp edges and corners. The areas with a sub-unity refractive index and the singular values at the corners are replaced with the refractive index of one (*n* = 1), which reduces the structure’s efficiency because these new unity-index regions do not affect the phase compensation [32,33,34].

In this study, we design a broadband lens with high gain and low reflection using conformal transformation optics. We try to provide an optimal structure to minimize the defects mentioned above. We select the structure’s corners in the physical space to differ slightly from those in the virtual space. Therefore, there are no singular values in the corners and no stretching in the structure’s top and lower boundaries. In this way, we minimize reflection with appropriate design. Proper design can reduce reflection but not eliminate it. We apply the optical path rescaling method to enhance the sub-unity refractive indices and to eliminate the reflection at the lens’s bottom boundary. The proposed flat lens antenna has the following advantages: isotropic material, low reflection, broadband, low side-lobe level, high gain, and is practically realizable.

## 2. Design Method

### 2.1. Design of Flat Lens Based on CTO

Transformation optics utilizes a proper coordinate transformation to connect two spaces, defined as virtual and physical spaces. If we consider Cartesian coordinates, the virtual and physical spaces are described as (*u*, *v, w*) and (*x*, *y*, *z* = *w*), respectively. Thus, the transformation is effectively applied in only two-dimensional (2D) space. The changes caused by the transformation coordinate are represented in the component properties of the media. The coefficients of permittivity and permeability in physical space (*ε′*, *μ′*) relate to these parameters in virtual space (*ε*, *μ*) as [1]

(1) $$\varepsilon^{\prime }=\frac{J\varepsilon J^{T}}{\det(J)},\;\mu^{\prime }=\frac{J\mu J^{T}}{\det(J)}$$

where *J = ∂(x,y,z)/∂(u,v,w)* is the Jacobian matrix, which in 2D can be written as

(2) $$J=\left[\begin{array}{ccc} \frac{\partial x}{\partial u} & \frac{\partial x}{\partial v} & 0\\ \frac{\partial y}{\partial u} & \frac{\partial y}{\partial v} & 0\\ 0 & 0 & 1 \end{array}\right]$$

Note that the virtual space is usually considered to be a vacuum. We can apply a conformal mapping by considering the complex variables *W* = *u* + *iv* and *Z* = *x* + *iy.* The analytical function *Z*(*W*) represents a conformal mapping between the virtual and physical spaces and leads to simple constitutive parameters of the material in the form of *ε′* = *µ′* = *diag* (1, 1, (*detJ*)^−1^). Therefore, the refractive index of physical space can be represented as

(3) $$n\left(x,y\right)=\sqrt{\varepsilon_{zz}\left(x,y\right)}=\sqrt{{\left(\det J\right)}^{-1}}=\frac{1}{\sqrt{x_{u}^{2}+x_{v}^{2}}}$$

**Figure 1:**
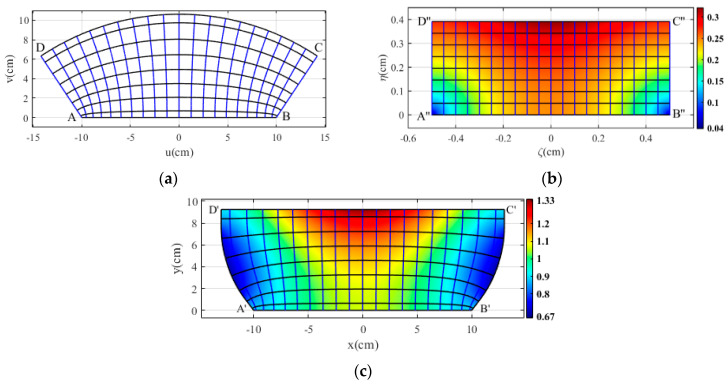
The flat lens design procedure, (**a**) virtual space, (**b**) intermediate space, and (**c**) physical space and refractive index of flat lens. Blue (vertical) and black (horizontal) lines represent equivalent contours in each space.

In the following, we use the CTO for designing the desired flat lens at the operating frequency of 15 GHz. The design procedure for the proposed flat lens is depicted in Figure 1. It can be seen that the virtual and physical spaces are represented as (*u*, *v*) and (*x*, *y*). We consider an intermediate space to map virtual to physical spaces, shown with (*ζ*, *η*) in Figure 1b. In the virtual space shown in Figure 1a, CD is an arc of a circle, and sides AD and BC align with the circle’s radius, yielding: AB = 20 cm, arc length CD = 30 cm, ∡C = ∡D = 90°, and ∡A = ∡B = 124°. The intermediate space is a rectangle with a length A″B″ = 1 cm and a width proportional to the design conformal module. The virtual space is mapped to physical space shown in Figure 1c with specifications A′B′ = 20 cm, C′D′ = 26 cm, ∡A′ = ∡B′ = 127°, and A′D′ = 9.25 cm. The lateral boundaries are curved rather than straight lines, such that the ∡D′ = ∡C′ = 90°.

If all quadrilaterals have the same conformal module, they are conformally equivalent [37]. The conformal module (*M*) is the rectangle’s aspect ratio in the intermediate space. However, *M* is obtained by solving a boundary value problem in virtual and physical spaces [10]. The virtual space is mapped to the physical space by having the coordinates of the three spaces and solving Laplace’s equations in two steps. In the first step, the virtual space is conformally mapped to the intermediate one. The Neumann and Dirichlet boundary conditions can be written as

(4) $$\begin{array}{l} {\xi |}_{AD}=-0.5\;;\;{\xi |}_{BC}=0.5\;\;\;\;\;\;\;\;\;\;;\overset{⌢}{n}.\nabla {\xi |}_{AB,DC}=0\\ {\eta |}_{AB}=0\;\;;\;{\eta |}_{DC}=1/M=0.39\;\;;\;\overset{⌢}{n}.\nabla {\eta |}_{AD,BC}=0 \end{array}$$

Therefore, the refractive index of intermediate space using (2), as shown in Figure 1b, is obtained as

(5) $$n(\xi ,\eta )=\frac{1}{\sqrt{\left(\xi_{u}^{2}+\xi_{v}^{2}\right)}}$$

In the next step, the physical space is conformally mapped to the intermediate one. In addition, the Neumann and Dirichlet boundary conditions can be written as

(6) $$\begin{array}{l} {\xi |}_{A^{\prime }D^{\prime }}=-0.5;\;{\xi |}_{B^{\prime }C^{\prime }}=0.5\;\;\;\;\;\;\;\;\;\;\;\;\;\;\;\;\;;\;\overset{⌢}{n}.\nabla {\xi |}_{A^{\prime }B^{\prime },D^{\prime }C^{\prime }}=0\\ {\eta |}_{A^{\prime }B^{\prime }}=0\;\;\;\;\;\;;\;{\eta |}_{D^{\prime }C^{\prime }}=1/M=0.39\;;\;\overset{⌢}{n}.\nabla {\eta |}_{A^{\prime }D^{\prime },B^{\prime }C^{\prime }}=0 \end{array}$$

where $\hat{n}$ is the normal vector to the boundaries and *M* = 2.55 is calculated by COMSOL. This process is the reverse of the preceding state, so *J = J^−1^*. Then, the final refractive index of physical space is calculated with the following formula

(7) $$n(x,y)=n(u,v)\times \sqrt{\left(\xi_{x}^{2}+\xi_{y}^{2}\right)}=\frac{\sqrt{\left(\xi_{x}^{2}+\xi_{y}^{2}\right)}}{\sqrt{\left(\xi_{u}^{2}+\xi_{v}^{2}\right)}}$$

The refractive index of the designed flat lens is represented in Figure 1c. All points have correspondence in all three spaces. As a result, the constant *η* and *ξ* lines in the intermediate space are mapped to corresponding lines in the virtual and physical spaces. These lines are illustrated in Figure 1 by black and blue lines. As shown, the refractive index values range from 0.67 to 1.33. There are no singular values at the corners because the vertices of physical space are almost similar to their equivalent in virtual space. There are sub-unity refractive index values, and the smallest values are related to the side boundaries. Furthermore, the refractive index is low at the lower boundary, but it does not match to free space, resulting in reflection. We utilize the optical path rescaling method to overcome these issues.

### 2.2. Refractive Index Control

The distance that the light follows in a space with a certain refractive index is called the optical path length. An optical path from point A to point B along the ray can be described as

(8) $$u=\int_{A}^{B}n\;dl$$

where *n* and *u* denote the refractive index and optical path length as a function of ray length, respectively. *A* is the point of the ray’s intersection with the upper boundary of the flat lens (*C′D′*), where we consider the optical path zero. Furthermore, Equation (7) can be rewritten as n (x,y)=|∇u|. We can consider an increasing function, such as *S*(*u*), and assume that *S*(*u*) measures the optical path rather than *u*. Thus, the refractive index, known as the rescaled refractive index (*n′*), is determined as

(9) $$n^{\prime }(x,y)=\left|\nabla S(u)\right|=\left|\frac{dS}{du}\right|n(x,y)$$

**Figure 2:**
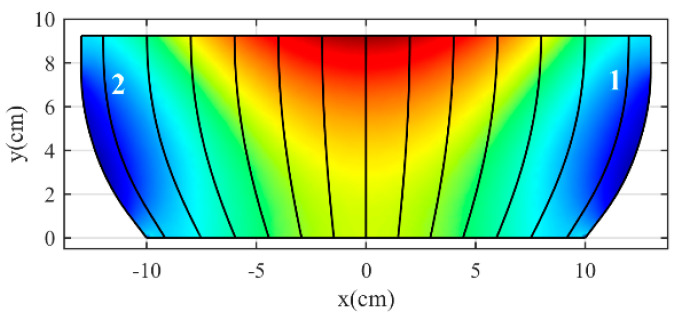
The chosen ray to utilize the rescaling refractive index method.

**Figure 3:**
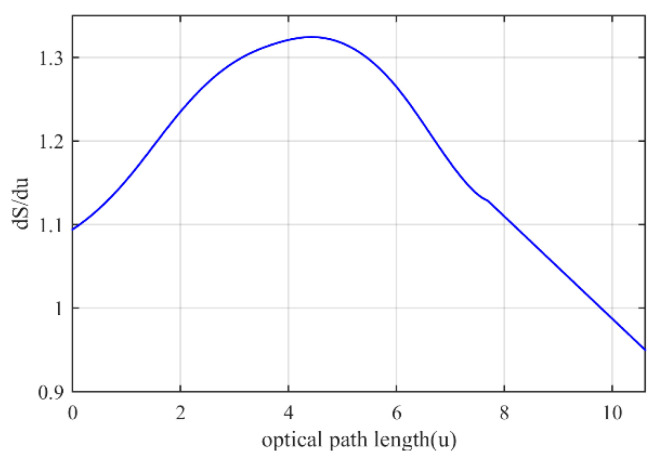
The derivative of the increasing function *dS*(*u*)/*du*.

Note that the refractive index varies, but the form of the rays and the phase-front remain constant. As a result, the structure’s performance remains unaffected. To apply this method, we must consider a proper ray. For this reason, the ray trajectories are plotted in physical space using the ray tracing in the COMSOL Geometrical Optics (GO) module with a located point source at a distance of 14.75 cm from the lens. Eventually, we select one of the two rays, as shown in Figure 2. This ray does not cover the entire length of the optical path, so we divide the optical path length into two parts. From *u*_0_ to *u*_1_, the increasing function is in the form of Equation (9). Moreover, to continue the path, we consider a quadratic equation connected to the previous function.

(10) $$S(u)=\int_{u_{0}}^{u_{1}}\frac{1}{n(u)}du$$

where *n*(*u*) is the refractive index versus the optical path along the selected ray. The derivative of both this function and the considered quadratic function is shown in Figure 3.

**Figure 4:**
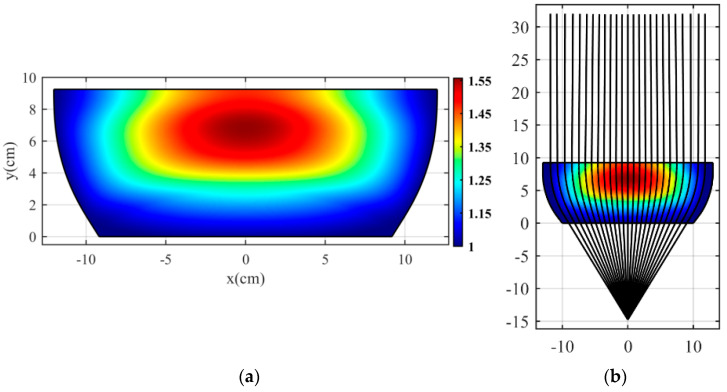
(**a**) Refractive index of designed flat lens after applying the optical path rescaling method, and (**b**) simulated ray trajectories in the COMSOL ray tracing module.

If we apply the *dS*/*du* to the whole physical space, we obtain the refractive index *n′*(*x*, *y*) seen in Figure 4a. Note that applying this function requires having the optical path for all physical space coordinates. To gather this information, we first use the ray tracing method to obtain the coordinates of 1100 rays and then interpolate in physical space to determine the refractive index for these coordinates. In this case, we place the source point at a distance of −14.75 cm from the physical space and consider the coordinates of ray trajectories inside the structure. Eventually, we compute the optical path numerically for all points using (7). Moreover, the selected ray for applying the optical path rescaling method does not have the lowest refractive index value. Consequently, the distance between the selected ray and the side boundary has a sub-unity refractive index, which covers a small part of the lens’s sides and does not affect its performance. As such, we remove these sections. Figure 4a indicates that all refractive index values less than one have disappeared, and the lower boundary of the designed flat lens matches to free space. Figure 4b confirms the proper performance of the proposed flat lens by showing the ray trajectories in the COMSOL ray tracing module. As shown in Figure 4b, the source point is placed at a distance of −14.75 cm from the designed flat lens.

## 3. Simulation Results

**Figure 5:**
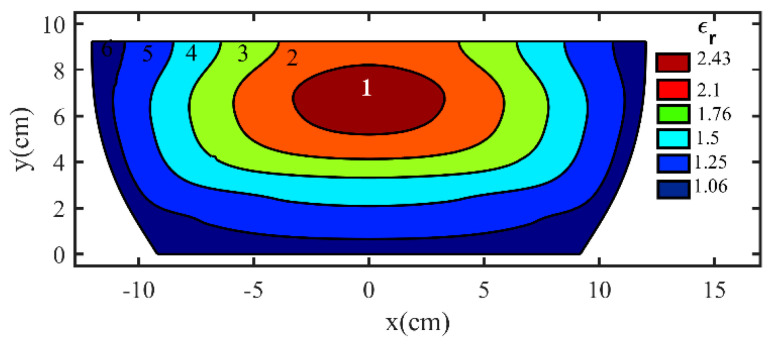
The designed flat lens profile consisting of 6 layers with the permittivity value in each layer.

**Figure 6:**
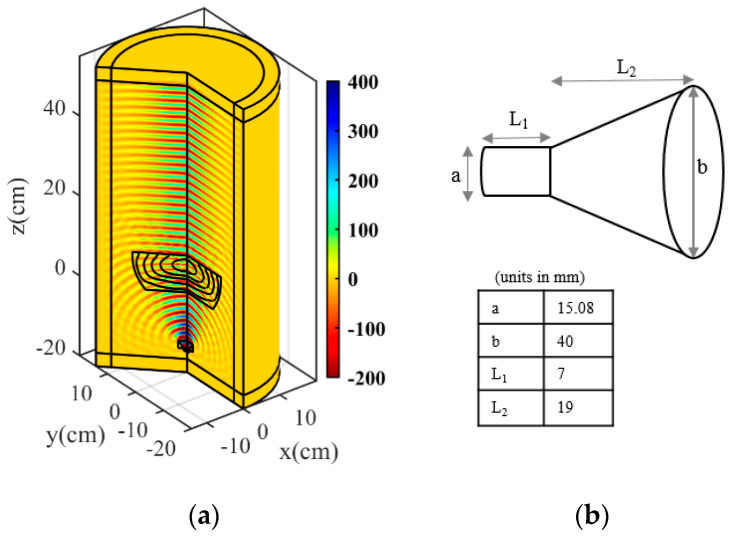
3D structure of proposed flat lens antenna, (**a**) electric field magnitude, and (**b**) feeding antenna with its dimensions in millimeters.

The permittivity profile of the proposed flat lens has been modeled using six layers ranging from 1.06 to 2.43. Figure 5 depicts the final two-dimensional structure and the relative permittivity coefficient in each layer. The final three-dimensional (3D) profile of the flat lens antenna is obtained by rotating the permittivity profile around the propagation direction. We utilized the 2D Axisymmetric in COMSOL Multiphysics to obtain the 3D profile. A feeding horn antenna is placed 14.75 cm away from the lens to evaluate the radiation properties of the proposed flat lens. Figure 6a depicts the 3-D flat lens in the COMSOL.

A conical horn connected to a standard Ku-band circular waveguide makes the feed antenna. The waveguide’s diameter and length are a = 15.08 mm and L1 = 7 mm, respectively. Moreover, the diameter and length of the horn are b = 40 mm and L2 = 19 mm. The feed antenna dimensions are shown in Figure 6b.

**Figure 7:**
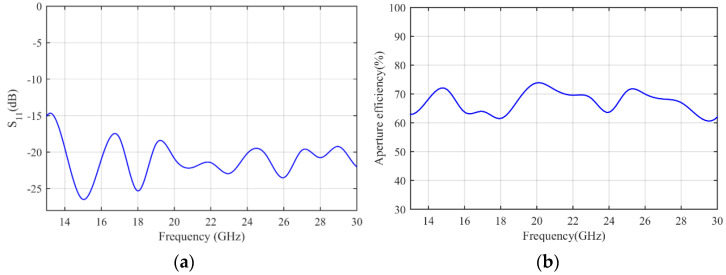
(**a**) Simulated S_11_ parameter, and (**b**) aperture efficiency of proposed flat lens antenna.

**Figure 8:**
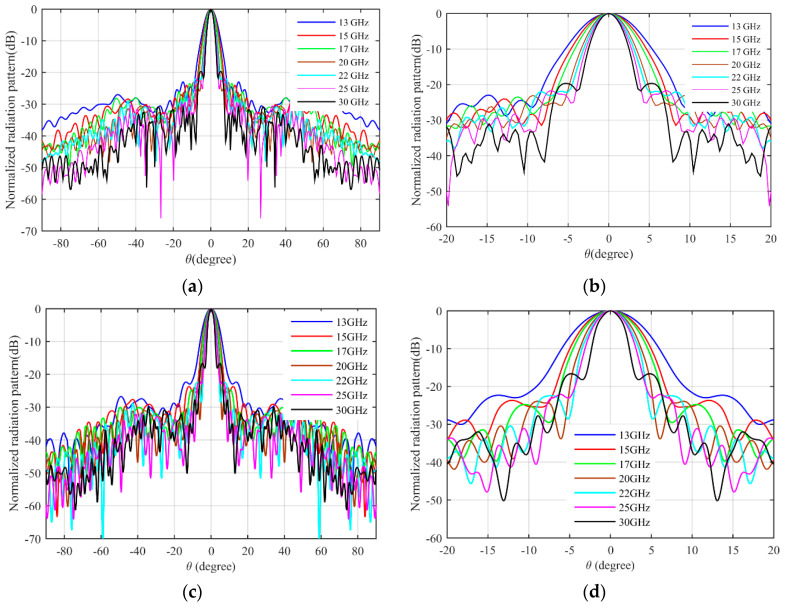
Simulated normalized radiation pattern of the proposed flat lens, (**a**) COMSOL results, (**b**) the corresponding magnified picture, (**c**) CST results, and (**d**) the corresponding magnified picture.

**Figure 9:**
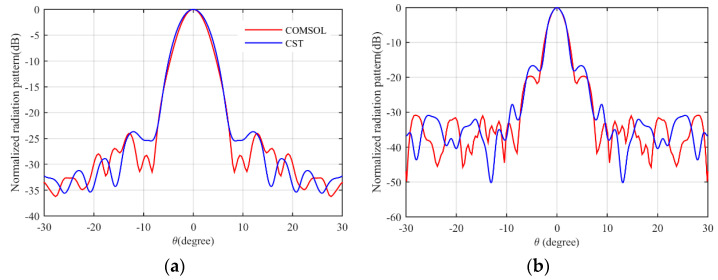
Comparison of COMSOL and CST radiation pattern simulations, (**a**) 15 GHz, and (**b**) 30 GHz.

**Figure 10:**
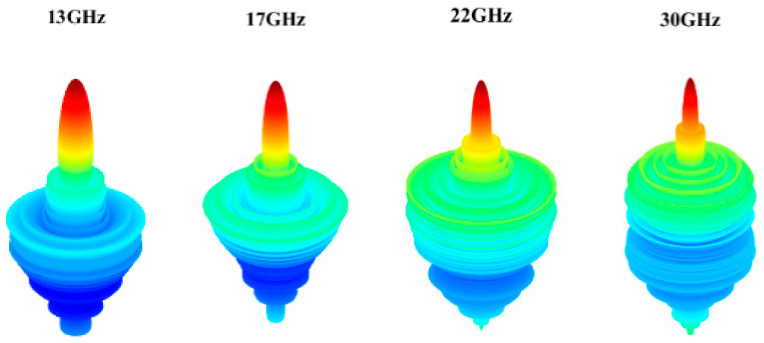
Simulated 3D radiation pattern of the proposed lens antenna at various frequencies.

**Figure 11:**
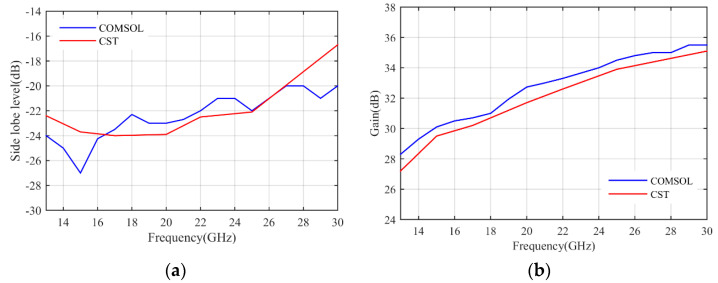
Comparison of COMSOL and CST Simulated results, (**a**) the side lobe level, and (**b**) the gain.

The simulation results are obtained by COMSOL Multiphysics. Figure 7a shows the S11 parameter of the feeding horn while illuminating the flat lens, as shown in Figure 6a. It can be seen that the S11 value is less than −15 dB in the frequency range of 13–30 GHz, which shows low reflection from the proposed lens. Figure 7b presents the aperture efficiency of the proposed lens, which indicates good aperture efficiency from 62% to 74%. Figure 8a,b shows the simulated normalized radiation pattern by COMSOL at frequencies of 13–30 GHz. The half-power beam width (HPBW) changes from 5.6° to 2.7° when the frequency increases from 13 to 30 GHz. To confirm the simulation results, we simulated the proposed lens with CST Studio software, and the radiation patterns are demonstrated in Figure 8c,d. The radiation pattern results of COMSOL and CST are compared at f = 15 GHz and f = 30 GHz in Figure 9. The comparison of COMSOL and CST results shows a good agreement between them, which proves the validity of the results. Furthermore, to demonstrate the radiation performance, 3D radiation patterns are shown in Figure 10. The COMSOL and CST results for the side lobe level versus frequency are depicted in Figure 11a. As shown, the side lobe level is less than −16.5 dB in all of the frequency ranges, which indicates good radiation performance of the structure. Figure 11b shows the COMSOL and CST results for the gain of the proposed flat lens, which indicates an increase in gain with increasing frequency, as expected. Finally, the suggested flat lens antenna can be realized by six milled dielectric layers with different accessible permittivity [34].

**Figure 12:**
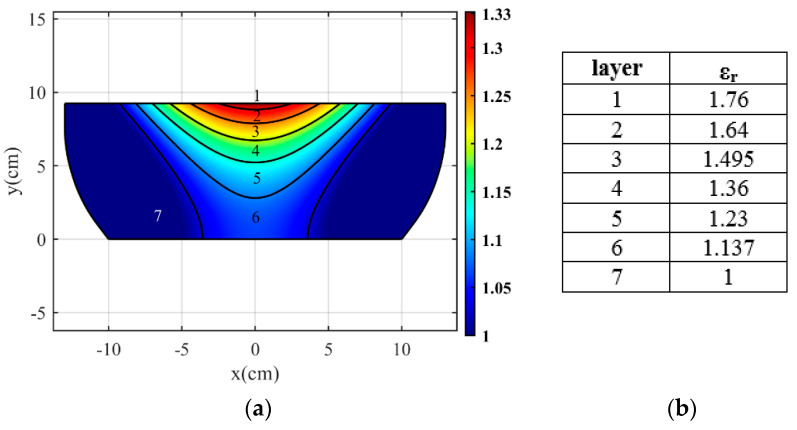
(**a)** The refractive index layers when the sub-unit values are replaced with unit value, and (**b**) the relative permittivity in each layer.

**Figure 13:**
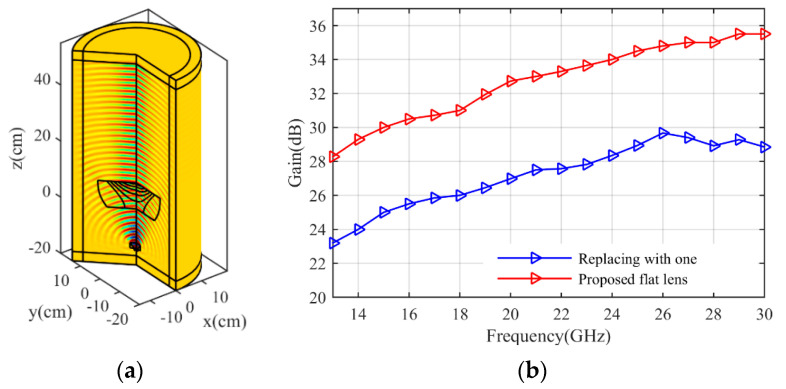
(**a**) 3D structure of the lens antenna, and (**b**) comparison of the lens gain with proposed flat lens gain.

**Figure 14:**
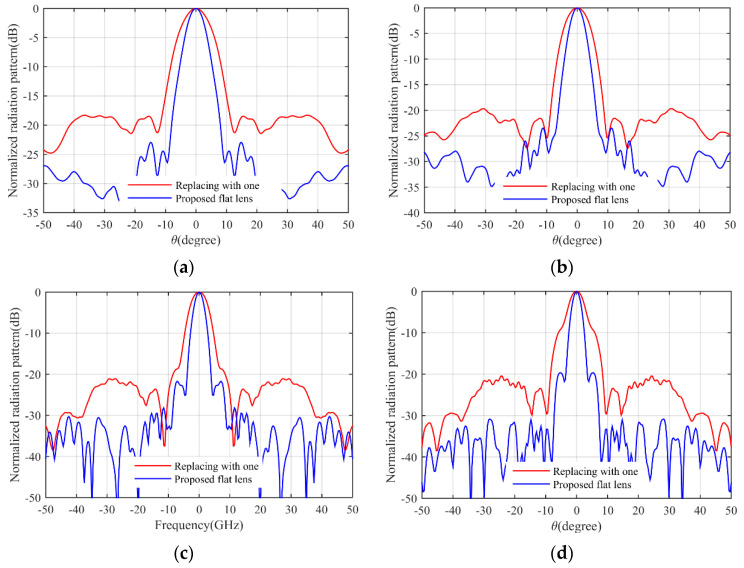
Comparison of the lens radiation pattern in different frequencies with the radiation pattern of the proposed flat lens, (**a**) 13 GHz, (**b**) 17 GHz, (**c**) 25 GHz, and (**d**) 30 GHz.

In previously designed flat lenses, the refractive index values of less than one have been replaced by one, which reduces the lens performance. To demonstrate this, we compare the simulation results of our proposed lens with the results when the sub-unit values are replaced by one. Suppose the sub-unit values in Figure 1c are replaced by one. The resulting refractive index of the structure is shown in Figure 12a. As can be seen, the continuous refractive index is divided into seven layers with the relative permittivity coefficients shown in Figure 12b. The three-dimensional structure in the simulation environment is depicted in Figure 13a.The gains for two cases (this lens and the proposed lens) are compared in Figure 13b. The gain increases from 23.2 dBi to 28.8 dBi. In addition, the radiation pattern of the lens is compared with the radiation pattern of the proposed lens in Figure 14 at different frequencies. As can be seen, the beam width and the side lobe level increase. As a result, implementing the proposed approach on previously designed lenses improves lens performance.

Table 1 summarizes the parameters of the proposed lens and recently published re-search in the literature for comparison. In previously designed flat lenses, the refractive index values of less than one have been replaced by one, which reduces lens performance. In this paper, we use the optical path rescaling method in the lens design, so the refractive index values below one are not removed, and the lens performance is not reduced. Furthermore, we reduce the reflection in the lower boundary of the proposed lens by using this method.

## 4. Conclusions

We designed a flat lens based on conformal transformation optics that leads to an implementable, isotropic, and all-dielectric material. Design considerations have resulted in singular refractive index values not being present in the proposed flat lens. Furthermore, we applied the optical path rescaling method to remove the sub-unit refractive index and reduce the reflection in the lower boundary of the lens. The final structure consists of six dielectric layers with constant permittivity value in each layer. The simulation results of the three-dimensional structure show the excellent performance of the proposed flat lens.

## Figures and Tables

**Table 1:** Comparison of results from this study with some recently published studies.

Reference	[19]	[33]	[35]	[36]	This Work
**Simulation software**	COMSOL	COMSOL	CST	CST	COMSOL and CST
**Method**	CTO	QCTO	QCTO	QCTO	CTO
**Implementation method**	Graded photonic crystal	8 layers with ε_r_ between 1–2.8	22 layers with ε_r_ between 1–3.2	9 layers with ε_r_ between 3–12	6 layers with ε_r_ between 1.06–2.43
**Frequency range**	14–22 GHz	7–13 GHz	8–12 GHz	27–40 GHz	13–30 GHz
**Size (Diameter, Height)**	D = 22.7 cm, h = 32 cm	D = 14.8 cm, h = 4 cm	D = 2.6 cm, h = 8.5 cm	D = 7.6 cm, h = 1.2 cm	D = 24 cm, h = 9.25 cm
**Gain**	33–35 dB	Not stated	17.5–22.5 dB (directivity)	23.4–25.5 dB	28.3–35.5 dB
**Side lobe level**	−24 dB	−11.5 dB	−14 dB	−10 dB	−20 dB
**HPBW**	12°–10°	Not stated	Not stated	6°–4°	5.6°–2.7°

## Data Availability

Not applicable.
